# Active exploration is important for reinforcement learning of interval timing

**DOI:** 10.1186/1471-2202-12-S1-P343

**Published:** 2011-07-18

**Authors:** Osamu Shouno, Hiroshi Tsujino

**Affiliations:** 1 Honda Research Institute Japan Co., Ltd., Wako, Saitama, 351-0188, Japan

## 

Timing of initiating action is often critical for performance of voluntary behaviors. Appropriate times for initiating voluntary actions are considered to be acquired by reinforcement learning. In this learning process, exploration in the time domain is essential. The basal ganglia have been implicated in the initiation of voluntary movements. Recently, we proposed a biologically plausible mechanism for probabilistic timing of action initiation in the basal ganglia, and by computer simulations of the spiking neural network model we demonstrated the probabilistic nature of the action initiation of the model which supports active exploration in the range of several seconds [1]. For further characterization of the probabilistic timing mechanism, we tested the simplified version of the model in numerical simulations of the interval generation task in which subjects are required to initiate an action after a specific period that follows a instruction stimulus. The simplified model is a leaky-integrator, which integrates incoming spikes and once the “membrane potential” reaches a threshold it generates an output. In each trial, a time point at which the model begins the input-integration is determined probabilistically based on the probability distribution which depends on the product of intrinsic “prior” probability distribution and a time course of total external inputs. The resultant distributions of output times resembled those of the spiking neural network model. We compared this active exploration model with the passive exploration model that is a similar leaky-integrator model but the input-integration begins at the instruction stimulus is presented. The input consisted of 5000 homogeneous Poisson spike trains of the same mean rate, and was fixed across trials in a session. The learning rule is as follows: a synapse that contributes to the generation of a desired output is strengthened while a synapse that contributes to the generation of an inappropriate output is weakened. In numerical simulations, as the mean rate of the input and/or the time interval between the appearance of the instruction stimulus and the target time progressively increased, the performance of the passive exploration model decreased dramatically. On the contrary, the active exploration model exhibits relatively stable performance in the same ranges of parameters. Heuristically, this drop in the learning performance in the passive model arises as a result of increasing conflicts on weight update of a synapse in proportion to increasing chances that a synapse could contribute both of desired and inappropriate outputs at different times. Because the passive model explores in a way that an output time is progressively increasing toward the target time by decreasing weights of synapses relevant to the generation of inappropriate outputs, the passive model tends to experience intensive decreases in weight of a synapse which should contribute to the desired output. However, the active exploration model explores much wider range of time than the passive one, and thus tends to avoid intensive deceases in strength of specific synapses. These results indicate the importance of the probabilistic timing of action initiation for reinforcement learning of interval timing.
